# Aligning emergency care with global health priorities

**DOI:** 10.1186/s12245-018-0213-8

**Published:** 2018-11-22

**Authors:** Thomas Shanahan, Nicholas Risko, Junaid Razzak, Zulfiqar Bhutta

**Affiliations:** 1 Thomas Shanahan, University of Leeds, Leeds, UK; 2 Department of Emergency Medicine, Johns Hopkins University School of Medicine, Baltimore, MD USA; 3 Aga Khan University, Islamabad, Pakistan

**Keywords:** Emergency care, Emergency care systems, Sustainable development goals, Low and middle-income countries, Universal health coverage, World health Assembly, World Health Organization

## Abstract

**Background:**

The availability of resources, knowledge, and will to expand access to high-quality emergency care in low- and middle-income countries has made strong progress in recent years. While the possibility for intervention has improved, the need has only grown more pressing. What remains is for us, the people who practice and support emergency care delivery on a regular basis, to pull these elements together and present a cohesive call to action for leaders to prioritize the development of emergency care. This advocacy should coalesce around two high-level commitments: the Sustainable Development Goals and Universal Health Coverage. Emergency care has not been a traditional tool that policy makers rely on to improve health and development; however, we can show that it is actually critical to achieving these goals. Making this case has become possible with the availability of evidence that shows emergency health conditions contribute to a substantial portion of the disease burden, emergency care interventions are high-impact, and the interventions can be implemented without a substantial increase in resources.

**Main body:**

There is a growing understanding of the burden of disease in low- and middle-income countries and how 54% or 24.3 million deaths are amenable to emergency care systems. There are a group of diseases that are time sensitive and show improved outcomes with good emergency care systems. Alongside an improving scientific underpinning to emergency care, there is growing policy recognition. While there is no direct mention of emergency care in the Sustainable Development Goals document, many goals, such as reductions in infant and maternal deaths, deaths due to non-communicable diseases, road traffic injuries and violence, improving resilience of climate change, universal coverage, and safe/sustainable urban environments are not achievable without developing, sustaining, and improving the quality of emergency care systems.

**Conclusion:**

To take emergency care to the next level, we must capitalize on the growing understanding of the disease burden of emergent conditions, along with the increasing evidence of the high-impact and low-cost of emergency care interventions. Linking these messages to widely accepted policy priorities like the SDGs and UHC will increase attention towards the development of emergency care systems, which potentially could save lives.

## Background

In May, leaders from around the globe convened in Geneva, Switzerland, for the 2018 World Health Assembly (WHA). This United Nations meeting is designed to identify the world’s top health challenges and align all member states on specific goals to address them.

Recent goals and long-term strategies that have emerged from these global discussions, include the health-related Sustainable Development Goals (SDGs) [1], a focus on Universal Health Coverage (UHC), and most recently the “3 Billions” 5-year World Health Organization (WHO) workplan [2].

An important remaining task is to pull these elements together and present a cohesive call to action for international community and stakeholders in low and middle-income countries (LMICs) to prioritize the emergency care provision and the development of emergency care systems. In fact, it has never been a better time to make the case for emergency care.

Although, emergency care has not been a traditional tool that policy makers rely on to improve health and development, growing evidence demonstrates that it is critical to achieving these goals. Making this case has become possible with the availability of evidence that shows emergency health conditions contribute to a substantial portion of the disease burden, emergency care interventions are high-impact, and that interventions can be implemented without a substantial increase in resources [3]. Below you will find a short discussion on our collective understanding of the value of emergency care in LMICs and how it fits into these global commitments.

## Improved understanding of the value of emergency care

As a result of The Global Burden of Diseases, Injuries, and Risk Factors Study (GBD), we now have a much better understanding of the nature of the emergency care challenges in LMICs. A recent study found “all leading 15 global causes of death and [Disability Adjusted Life Years] DALYs were conditions with potential emergent manifestations” [4]. Emergent manifestations were defined as “conditions that, if not addressed within hours to days of onset, commonly lead to serious disability or death [and] conditions with common acute decompensations that lead to serious disability or death” [4]. In fact, all the five most frequent causes of death in LMICs—ischemic heart disease, stroke, lower respiratory infections, chronic obstructive pulmonary disease, and diarrheal diseases—can present as emergencies, can be time sensitive, and show improved outcomes with quality emergency care. The same is true for maternal and neonatal deaths and injuries [5]. The evidence suggests over half the deaths in low-resource settings could be addressed by improvements in emergency care [5].

There is evidence showing triage, trained lay first responders, paramedic responders with basic life support (BLS) training, better flow in hospitals, and supervision of junior providers reduces mortality in low-resource settings [5]. Studies from developed countries have shown the public health impact of emergency departments through prevention, screening, and early referral for a wide variety of conditions such as HIV, cardiovascular diseases, drug abuse, smoking, hypertension, domestic violence, increase use of seat belts, and helmets [6].

Early data has shown that emergency care interventions can be cost-effective [7, 8]. Often the simple reorganization of service delivery can leverage existing health system resources to improve outcomes without increasing costs, such as triage interventions. Basic emergency care training interventions can produce extraordinary gains at little cost, for example, one study has shown trained lay responders cost “$170 per death averted, and $7 per life year gained for a population of 1 million” [7].

## Linking emergency care to global health priorities

With this growing evidence, the moment has arrived to integrate emergency care development into established global health priorities (see Table 1 below). With the adoption of the World Health Assembly (WHA) Resolution 60.22 Health Systems: Emergency Care Systems [9] in 2007, there has been some recognition amongst policy makers of the importance of emergency care. This has been enhanced in 2015 by the World Health Assembly Resolution 68.15 on strengthening emergency and essential surgical care and anesthesia as a component of universal health coverage [10]. The release of the WHO Emergency Care System Framework, which provides a visual understanding of the essential emergency care functions, along with other emergency care specific guidance tools, provides a shared language for health professionals and policy makers to discuss what emergency care systems should look like and evaluate where improvements can be made [11]. Despite many important markers of progress, there is no direct mention of emergency care in relation to either of the leading global health priorities for the upcoming decade, the SDGs and UHC.

**Table 1:** Selected global health priorities related to emergency care

Sustainable Development Goals (SDG) (2015–2030)	- 17 development targets agreed upon by all United Nations member states - Targets related to emergency care include: 3.1–3.9, 11, and 16
World Health Organization 5-year workplan (2019–2023)	- Endorsed by the World Health Assembly in May, 2018 - Transforms the World Health Organization around 3 major goals: ○ 1 billion more people benefitting from Universal Health Coverage ○ 1 billion more people protected from health emergencies ○ 1 billion more people enjoying better health and well-being
Universal Health Coverage (UHC) (2015–2030)	- Incorporated into the SDGs (3.8) and the WHO workplan - UHC enables everyone to: ○ Access high-quality services that address the most important causes of disease and death ○ Be protected from the financial risk of accessing those services

Many SDG goals are not achievable without developing, sustaining, and improving the quality of emergency care systems (see Table 1). Specifically, emergency care is critical for treating obstetric emergencies, such as hemorrhage and sepsis and acute pediatric conditions, such as sepsis, diarrhea, and pneumonia, as well as the acute complications of communicable diseases, substance misuse, and acute exposure to hazardous materials. Well-functioning emergency care is critical for preventing road traffic accidents and violence and providing care to people with injuries. Generally, reductions in infant and maternal deaths and deaths due to non-communicable diseases (NCDs), road traffic injuries and violence, improving resilience of climate change, universal coverage, and safe/sustainable urban environments are not achievable without emergency care systems. Developing emergency care systems and research and surveillance capabilities are key to preparedness for and response to disasters, conflict, and public health emergencies in LMICs.

The WHO understands UHC to mean that all individuals and communities receive the health services they need without suffering financial hardship. This requires removing any barriers to equitable access to health services across the population. It also necessitates the development of protection against unexpected, catastrophic health spending that can create or worsen impoverishment. While a range of health services contribute to providing UHC, emergency care stands out as critical to its final achievement. Emergency care is unique in that it serves as a safety net and last resort for populations who are unable, unwilling, or unlikely to access preventive or primary care services. In many settings, emergency care is the only medical care that is provided regardless of ability to pay. For these reasons, a functioning emergency care system is required to achieve the UHC goals of access and financial protection.

In the recent WHA, a program of work for the next 5 years has been set forth as a set of interconnected priorities and goals to ensure healthy lives and promote well-being. They surround three “1 billion” targets: 1 billion more people enjoying better health and well-being, 1 billion more benefitting from universal health coverage and 1 billion more better protected from health emergencies. Although it has remained unstated, it is quite clear that a well-organized emergency care system will be required for any country to make progress on these goals. The emergency care system plays integral roles in health, disasters, and the issue of UHC. One could imagine a small circle in the middle of these three interlinked circles representing the emergency care system as a unifying platform linking these three targets.

## Conclusions

All UN member states have made a policy commitment to achieving the SDGs by 2030. Progress on numerous health-related goals has been difficult, and country leaders are actively looking for solutions. The WHO, prompted by the votes of its member states, has also made a long-term commitment to the global expansion of universal health coverage beyond what is mentioned in the SDGs. Improving emergency care is key to meeting these high-level commitments to the SDGs and UHC. Messaging around this should emphasize the high-impact of emergency care interventions on the diseases prioritized in the SDGs and the unique role emergency care systems play in supporting UHC and implementation of the 3-billion workplan.

Emergency care practitioners should be advocating about the high impact of emergency care interventions in LMICs to influence the review of SDGs in 2019, especially goals 10 and 16 and in preparation for the 72nd World Health Assembly in May 2019.

## Abbreviations

BLS: Basic life support
DALYs: Disability-adjusted life years
GBD: Global Burden of Disease, Injuries, and Risk Factors Study
LMICs: Low and middle-income countries
NCDs: Non-communicable diseases
SDGs: Sustainable Development Goals
UHC: Universal Health Coverage
UN: United Nations
WHA: World Health Assembly
WHO: World Health Organization
